# Using Quality Improvement to Change Testing Practices for Community-acquired Pneumonia

**DOI:** 10.1097/pq9.0000000000000105

**Published:** 2018-09-20

**Authors:** Amanda J. Rogers, Patricia S. Lye, Daisy A. Ciener, Bixiang Ren, Evelyn M. Kuhn, Andrea K. Morrison

**Affiliations:** From the * Department of Pediatrics, Medical College of Wisconsin, Milwaukee, WI; † Department of Pediatrics, Vanderbilt University School of Medicine, Nashville, TN; ‡ Children’s Hospital of Wisconsin Milwaukee, MI.

## Abstract

**Background:**

National guidelines for pediatric community-acquired pneumonia (CAP) contain recommendations regarding diagnostic testing including chest radiographs (CXRs), complete blood counts (CBCs), and blood cultures. Local data indicated that our institution was not delivering care at standards outlined by these guidelines. This project aimed to decrease CXRs for children with CAP discharged from the emergency department (ED) by 10% and decrease CBCs and blood cultures for patients hospitalized with uncomplicated CAP by 20% within 1 year.

**Methods:**

This single-site quality improvement initiative targeted otherwise healthy children 3 months to 18 years who presented to the ED with uncomplicated CAP at a free-standing academic children’s hospital. A quality improvement team performed a series of interventions including guideline implementation, data sharing, and annual education. Process measures included CXR, CBC, and blood culture rates. Balancing measures included the number of patients diagnosed with CAP, the frequency of antibiotic use, length of stay, and ED and hospital return rates. The team used statistical process control charts to plot measures.

**Results:**

There was special cause improvement with a desirable downward shift in testing that correlated with the project’s interventions. The percentage of CXRs for discharged patients decreased from 79% to 57%. CBCs and blood cultures for hospitalized patients decreased from 30% to 19% and 24% to 14%, respectively. Balancing measures remained unchanged.

**Conclusions:**

We used elements of quality improvement methodology to reduce testing for uncomplicated CAP without impacting the number of patients diagnosed with CAP, the frequency of antibiotic use, length of stay, and reutilization rates.

## Abstract 2

Supplemental Digital Content is available in the text.

## INTRODUCTION

Community-acquired pneumonia (CAP) is one of the most common diagnoses for pediatric hospitalization outside of the newborn period.^1^ Studies have shown considerable variation in diagnostic testing for CAP.^2,3^ CAP is, therefore, an ideal target for quality improvement (QI) efforts.

In 2011, the Infectious Disease Society of America (IDSA) released guidelines for the management of children with CAP^4^ that includes testing recommendations: The IDSA guidelines recommend chest radiographs (CXRs) for patients hospitalized with CAP but not for patients treated as an outpatient. Complete blood count (CBC) and blood culture recommendations vary based on severity. The IDSA guidelines recommend CBCs for patients hospitalized with severe CAP and blood culture for patients hospitalized with moderate-to-severe CAP, particularly those with severe or complicated CAP. These recommendations suggest less testing than was current practice at our institution where over three-fourths of patients with pneumonia treated as outpatients underwent CXRs and a quarter of all patients had CBCs and blood cultures obtained. Overtesting has the potential to lead to unnecessary pain from venipunctures, radiation exposure from CXRs, excess costs,^5^ and additional interventions without necessarily improving patient care.^6^ There was an opportunity to engage providers to align with standard recommendations to decrease unnecessary testing.

The overall goal of this project was to decrease unnecessary testing to comply with standard care for patients with uncomplicated CAP. The specific aims were (1) to decrease CXRs obtained in the emergency department (ED) for patients treated as an outpatient by 20%; (2) to decrease CBCs obtained in the ED for hospitalized patients by 10%; and (3) to decrease blood cultures obtained in the ED for hospitalized patients by 10% all within 1 year.

## METHODS

### Context

Faculty from the divisions of Pediatric Hospital Medicine (PHM) and Pediatric Emergency Medicine (PEM) saw an opportunity to improve practice. The PHM and PEM faculty worked jointly due to closely connected practices in caring for these patients. Both divisions have active QI groups and processes for developing guidelines, but there was no infrastructure for joint work. There was an accessible location for guidelines for PHM on the Children’s Hospital of Wisconsin (CHW) intranet and PEM in the electronic health record (EHR) (EPIC) drop-down menu. CHW is a teaching hospital with multiple levels of learners. It was standard practice for learners to utilize guidelines and to participate in QI work.

Faculty from both divisions see patients at CHW, a free-standing tertiary-care children’s hospital and level 1 trauma center associated with an academic medical center. It has 306 beds, over 65,000 ED visits annually, and over 20,000 admissions annually. CHW is the only children’s hospital in southeastern Wisconsin. Approximately, 93% of all pediatric hospital admissions in the greater Milwaukee metropolitan area are to CHW.

#### Population.

**Fig. 1.**
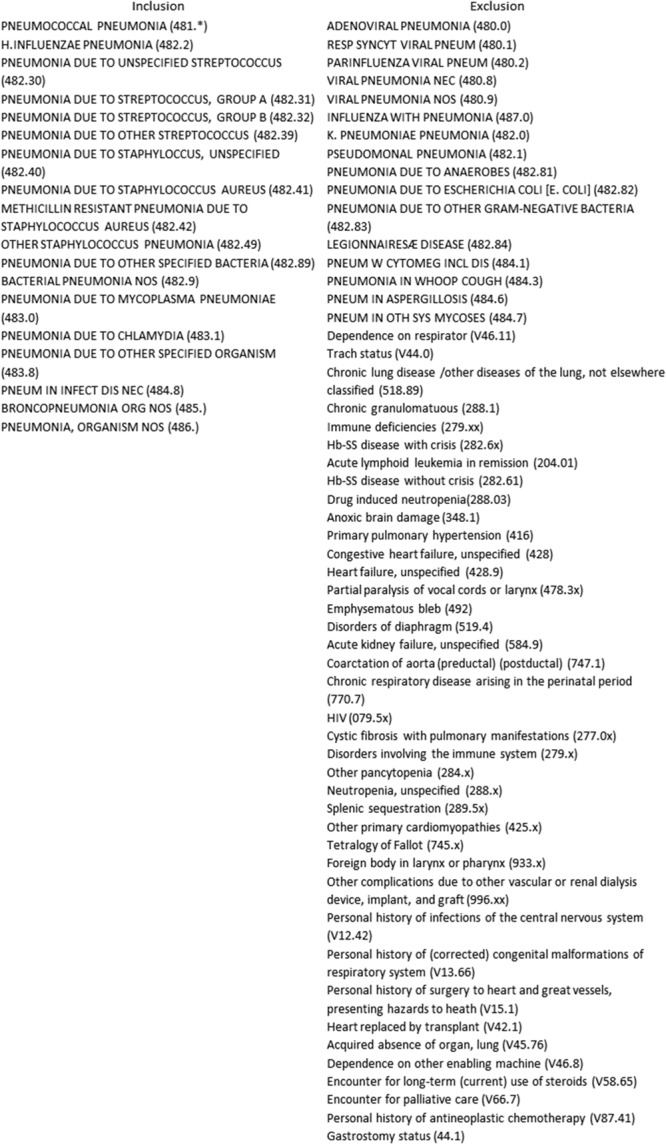
ICD-9 inclusion and exclusion criteria.

The population included otherwise healthy children 3 months to 18 years who presented to the CHW ED with uncomplicated CAP. The study included patients with a primary or secondary discharge diagnosis code (International Classification of Diseases, ninth revision [ICD]-9) of pneumonia that providers discharged from the ED or admitted to the noncritical care service. The diagnosis of CAP was not standardized but relied on the provider’s clinical decision and included patients with underlying asthma or reactive airway disease. The study excluded patients with an effusion, a primary or secondary diagnostic code consistent with a chronic disease, an immunocompromised state, chronic lung disease other than asthma, and those admitted to critical care (Fig. 1).

#### QI Team.

The team consisted of PHM and PEM physicians, a project coordinator, a QI specialist, a data analyst, and an EHR analyst. The team consulted other services for input including Infectious Disease, Critical Care, and Respiratory Care services.

### Interventions

We conducted Plan-Do-Study-Act cycles to implement and evaluate interventions.

#### Intervention 1: Local Guideline Development and Implementation.

The first intervention was the development and implementation of a local guideline for the management of uncomplicated CAP that was an adaptation of the IDSA guidelines. The process began with the formation of a joint PHM and PEM workgroup in June 2013 who assessed baseline data for testing practices to aid in the development of the local guideline and conducted a literature review. EHR analysts provided baseline data to identify areas to target where local practice differed from national guidelines. Baseline data showed that the local rates of CXRs for outpatient and CBCs and blood cultures in hospitalized patients were high. We therefore identified these measures as areas for improvement. On the other hand, rates of c-reactive protein and erythrocyte sedimentation rates, blood cultures for outpatients, and CXRs for inpatients were appropriate based on national guideline recommendations. We therefore did not target these for improvement.

The study team also used baseline data to guide local recommendations when the IDSA guideline were open to interpretation. For example, the IDSA guideline recommendations for laboratory testing is based on severity. It states that CBCs should be obtained in severe CAP and blood cultures should be obtained in moderate-to-severe CAP. The IDSA guidelines do not provide well-defined definitions for those severity levels. Which patients to test is therefore left to the interpretation of these classifications. Our local baseline data showed that blood cultures rarely positively impacted patient care in noncritical care patients hospitalized with uncomplicated CAP and that blood cultures often led to negative consequences in those patients due to the need for additional testing and increased length of stay (LOS) related to false positives. The study team, therefore, chose to recommend not obtaining CBCs or blood cultures in noncritical care patients with uncomplicated CAP.

During a multidisciplinary conference in December 2013 for PHM and PEM providers, the team outlined the proposed new local guideline, rationale, and baseline data. The conference was interactive, and the guideline was open for comment. Conference attendees set the initial targets for improvement based on baseline data and expert opinion. For example, the target to reduce the chest x-rays was relatively lower than that of blood testing, because there was more hesitancy of the attendees to reduce chest x-rays. After the conference, we adjusted the guideline based on attendee input.

The resulting guideline (available as **Supplemental Digital Content 1** at http://links.lww.com/PQ9/A40) incorporated recommendations regarding testing including not obtaining CXRs in patients with CAP well enough to be treated as an outpatient and not obtaining CBCs or blood cultures in patients with uncomplicated CAP hospitalized in the noncritical care setting. We sent the guideline via e-mail to all members of the divisions and placed it on a PHM intranet page and the PEM EHR drop-down menu.

#### Intervention 2: Provider Feedback Regarding Testing Rates.

Following the multidisciplinary conference and guideline dissemination, there was initial decreased testing for patients with CAP. However, over time it was noted that testing again increased. With provider turnover and multiple additional initiatives, the initial impact waned. The study team, therefore, planned a subsequent intervention of reviewing current data at monthly PHM and PEM section meetings. This change provided a forum for direct feedback to providers on testing rates and an opportunity to answer questions related to the guideline.

#### Intervention 3: Annual Re-education.

To ensure sustained change, we implemented an annual re-education session each fall before peak pneumonia season with data updates for providers and residents. In August 2015, we presented this information at Grand Rounds, which is a formal weekly conference for faculty, community pediatricians, and residents.

### Measures

#### Process Measures.

Process measures included the percentage of all patients discharged from the ED with the diagnosis of CAP who had a CXR obtained in the ED and the percentage of all patients hospitalized with CAP who had a CBC or blood culture obtained in the ED.

#### Balancing Measures.

Balancing measures related to CXRs included the number of patients discharged from the ED with a diagnosis of uncomplicated CAP and the percentage of all patients with uncomplicated CAP discharged from the ED who had antibiotics ordered. These measures were meant to assess any potential change in the number of patients diagnosed with or treated for CAP as a result of not obtaining CXRs. Another balancing measure related to CXRs was the percentage of patients with uncomplicated CAP discharged from the ED who returned to the ED within 48 hours and 7 days. This assessed for increased cases of inappropriate management caused by not obtaining a CXR that led to representation to the ED.

Balancing measures related to CBC and blood culture use included LOS and 48-hour and 7-day hospital readmission rates. These measures assessed whether not obtaining these tests led to inadequate treatment that resulted in prolonged courses or readmissions.

To better understand the role of blood cultures in the population, the team performed a manual review of all positive blood to assess if those represented true positive pathogens or potential contaminants. This distinction was determined based on the documented assessment and management of the positive culture. If the provider documented that the culture was clinically relevant or adjusted management based on the result, it was considered to be a true positive. If the provider documented that the culture was felt to be a contaminant or did not adjust management based on the result, it was considered to be a false positive.

### Analysis

We collected data between November 2012 and October 2015. We selected this period due to the implementation of a new EHR in November 2012 that allowed for data collection and the transition to ICD-10 in November 2015. The latter would have adversely impacted the accuracy of the data as the inclusion criteria were based on ICD-9 codes.

We obtained data through a systematic data pull performed by the EHR specialist and entered into a data repository (EPIC iNSIGHT).^7^

Statistical process control p-charts tracked the measures over time. P-charts, X-Bar charts, and C-charts tracked balancing measures. Standard tests^8^ to distinguish special (or assignable) cause variation from common cause (or random) variation were applied.

To ensure data reliability, we reviewed ICD-9 codes for secondary diagnoses to determine which to exclude based on the inclusion/exclusion criteria. We also performed a manual review of the charts associated with points outside the control limits and removed any that did not meet the inclusion criteria.

CHW Institutional Review Board approved the study.

## RESULTS

Over the course of this initiative, 1,901 patients were seen in the ED and diagnosed with pneumonia. Providers admitted 522 of those patients to acute care and discharged 1,379 from the ED.

### CXRs for Outpatients

**Fig. 2.**
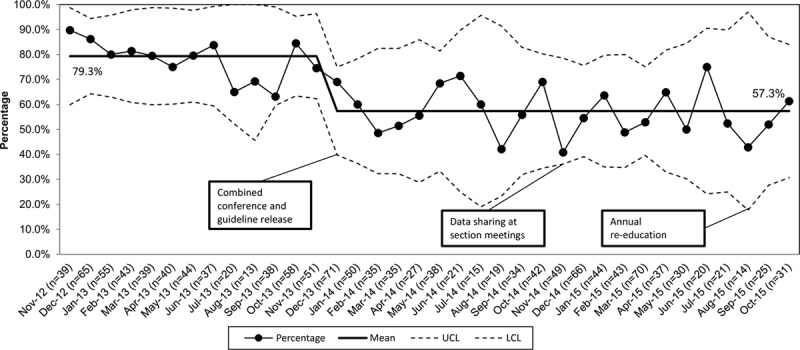
P-chart: percentage of discharged pneumonia patients with CXR ordered. P-chart for primary process measure, percentage of pneumonia patients providers discharged from the ED after having a chest x-ray ordered during the encounter. LCL, lower control limit; UCL, upper control limit.

The mean percentage of CXRs obtained at baseline for patients discharged from the ED was 79% and after guideline implementation was 57%, an absolute improvement of 22%. The P-chart (Fig. 2) revealed a desirable downward shift, which met the criteria for special cause variation following guideline implementation. We sustained this change for 22 months through the end of the data collection period.

For the balancing measures related to CXRs, there was no special cause variation in the number of patients diagnosed with uncomplicated pneumonia that providers discharged with an antibiotic or who returned to the ED within 48 hours or 7 days. The number of patients diagnosed with pneumonia varied with time of year as expected. However, the overall annual total number of patients diagnosed was stable year to year.

### Laboratory Testing

**Fig. 3.**
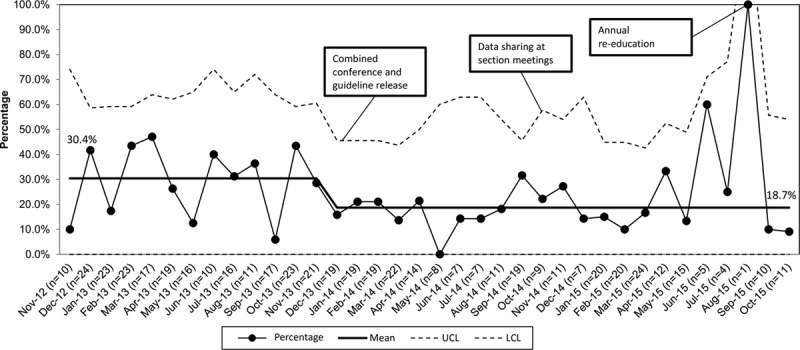
P-chart: percentage of admitted patients with complete blood count ordered. P-chart for primary process measure of the percent of pneumonia patients admitted from ED with a complete blood count ordered in the ED. LCL, lower control limit; UCL, upper control limit.

**Fig. 4.**
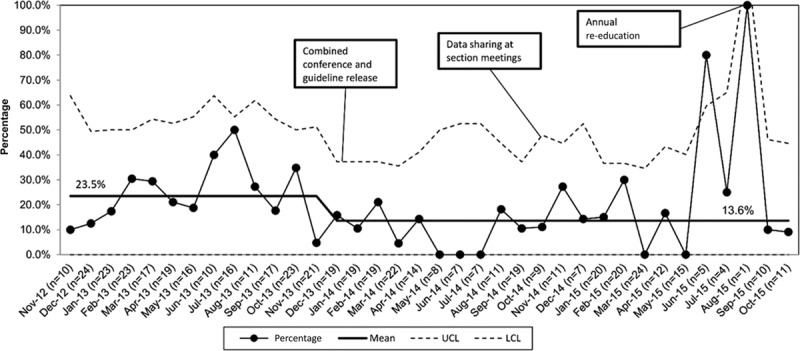
P-chart: percentage of admitted patients with blood culture ordered. P-chart for primary process measure of the percentage of pneumonia patients admitted from ED with a blood culture ordered in the ED. LCL, lower control limit; UCL, upper control limit.

The mean percentage of CBCs obtained at baseline for hospitalized patients was 30% and after guideline implementation was 19%, an improvement of 11% (Fig. 3). Providers obtained blood cultures in 24% of patients at baseline and 14% after guideline implementation, an improvement of 10% (Fig. 4). We saw a desirable shift meeting the criteria for special cause after guideline implementation for both CBC and blood culture testing.

During June 2015, the proportion of patients with blood cultures increased with special cause variation noted. After re-education in August 2015, this measure decreased back below the mean.

For the balancing measures related to CBCs and blood cultures, we did not note any change in LOS or 48-hour and 7-day return visit.

The study team completed chart reviews on all positive blood cultures to ensure the local guideline recommendation against obtaining blood cultures in patients hospitalized with uncomplicated CAP continued to represent the best practice for patients. Ninety-two of the 522 (17.6%) patients hospitalized with uncomplicated CAP had blood cultures obtained. Of those, 6 (6.5%) were positive. Of those 6, 4 were contaminants, and 2 were true positives. For the 2 true positives, both patients subsequently developed complicated CAP with parapneumonic effusions. One of those 2 patients required chest tube placement and a culture of the pleural fluid provided the same speciation and susceptibility information as the blood culture. For the 4 contaminants, 3 negatively impacted care by leading to repeating cultures, temporary broadening antibiotic coverage, and/or prolonging the LOS.

## DISCUSSION

### Summary

This QI initiative successfully reduced testing for uncomplicated CAP. Through interventions of guideline implementation, data sharing, and provider education, it achieved aims of decreasing CXRs for uncomplicated CAP treated as an outpatient and CBCs and blood cultures for uncomplicated CAP requiring hospitalization. These improvements have been sustained for nearly 2 years indicating a change in practice. The results did not identify negative impacts on the number of patients diagnosed with pneumonia, the frequency of antibiotic use, LOS, or reutilization rates.

The project was unique in the focus of the aims. Previous studies have examined improved compliance with the IDSA CAP guidelines. However, most focused on antibiotic selection rather than testing.^9–12^ Also, prior studies that did focus on testing aimed to increase testing, specifically with regard to cultures, rather than decrease testing, which was the aim of the project.^13^ To our knowledge, this is also the first study to successfully reduce CXRs for outpatients as recommended by the IDSA. Prior studies have found no reduction in CXRs after guideline release.^14^

Our project was also unique in that interventions were primarily education-based rather than a combination of education and hard-wired solutions as has been done in previous studies.^15^ The success of this initiative is likely due to successful knowledge translation practices implemented including the process of establishing a local consensus early, interactive discussion-based education meetings, and the process of audit and feedback to continue to improve performance.^16^ It is not always possible to implement hard-wired solutions. Pneumonia can present with nonspecific symptoms, and the testing targeted is used for many other disease states. This factor makes it difficult to target solely with hard-wired solutions such as best practice alerts. Also, not all EHR’s are adaptable to implement hard-wired solutions so educational-based interventions may be more generalizable. Education based interventions often require more effort to sustain, however, which we noted throughout this initiative. Moving forward consideration will be given to hard-wired solutions such as EHR prompts regarding testing recommendations to supplement the current education.

Finally, the study assessed balancing measures including reutilization rates and LOS. With growing attention on decreasing unnecessary health care utilization, the ability to reduce testing without negatively impacting patient care was notable.

### Interpretation

The results suggest that the interventions led to improvement in the process measures. We saw a desirable special cause variation in response to the main interventions for proportions of CXRs, CBCs, and blood cultures. Rates following intervention implementation were below national averages. National rates of CXRs for outpatients with pneumonia remain high at over 75% compared with the local rate of 57%. Similarly, national rates for CBC and blood cultures are reported to be 30–60%, again notably higher than the local rates of less than 20.^14^

The results did show an increase in blood cultures during June of 2015. This increase may be related to the fact that the summer is temporally far from the annual fall education, so providers may not readily recall specific recommendations or may be new to the institution since the last session. Also, there is a lower total number of patients with CAP that time of year due to the seasonal variability of CAP. Re-education the subsequent fall along with increasing volume of patients with CAP in winter months was associated with decreased testing in compliance with the guideline recommendations.

Prior studies have aimed to increase blood cultures^17,18^ for patients hospitalized with uncomplicated CAP.^15^ Local baseline and published data^19–21^ indicate that blood cultures rarely positively impacted care in noncritical care patients hospitalized with uncomplicated CAP. The local guidelines, therefore, recommended not obtaining blood cultures in those patients. Despite a decrease in blood cultures obtained, the results did not suggest an increase in LOS or reutilization, suggesting that there was not a meaningful increase in missed cases of clinically significant bacteremia. Also, of the blood cultures that providers obtained, most were clinically insignificant or led to unnecessary testing or treatment, which further supports not obtaining blood cultures unless otherwise indicated. These findings further justify the recommendation by other studies that in previously healthy children with nonsevere CAP, a blood culture does not add to the clinical care.^10,18^

### Limitations

Due to transitions in the local EHR, the analysis lacked baseline data from before the release of the IDSA guidelines. However, it is reasonable to suspect that baseline practice was to test more frequently before the release of national guidelines, and there was still noted improvement despite not knowing this earlier baseline.

Second, the reutilization rates include only return visits to this institution and may miss patients who re-present at other facilities. However, since the included patients initially presented to this institution, and since CHW is the only free-standing children’s hospital in the area with approximately 93% of all pediatric hospital admissions in the greater Milwaukee metropolitan area, these results capture the majority of returning patients.

Third, this is a single-center study, and the results may not be generalizable to other institutions.

## CONCLUSIONS

Using components of QI methodology, this initiative successfully developed and implemented an evidence-based guideline for the management of pediatric patients with uncomplicated CAP. This process led to a lasting reduction in ancillary testing for patients with uncomplicated CAP in alignment with national recommendations without leading to an inadvertent increase in clinical diagnosis, antibiotic use, LOS, or readmission rates. With a reasonable amount of annual re-education, these improvements have been sustainable for nearly 2 years.

## ACKNOWLEDGMENTS

Assistance with the study: the authors thank Mary Beth Miranda, Dr. Craig VanderWyst, Tom O’Neil, Lara Sobek, Maureen Collins, and Rob Thielke for their assistance with this project.

## DISCLOSURE

The authors have no financial interest to declare in relation to the content of this article.

## Supplementary Material

SUPPLEMENTARY MATERIAL
