# Supplementary figures and images for: Using Quality Improvement to Change Testing Practices for Community-acquired Pneumonia
Source: Pediatr Qual Saf. 2018 Sep 20;3(5):e105. doi: 10.1097/pq9.0000000000000105 (PMC6221590; doi:10.1097/pq9.0000000000000105)

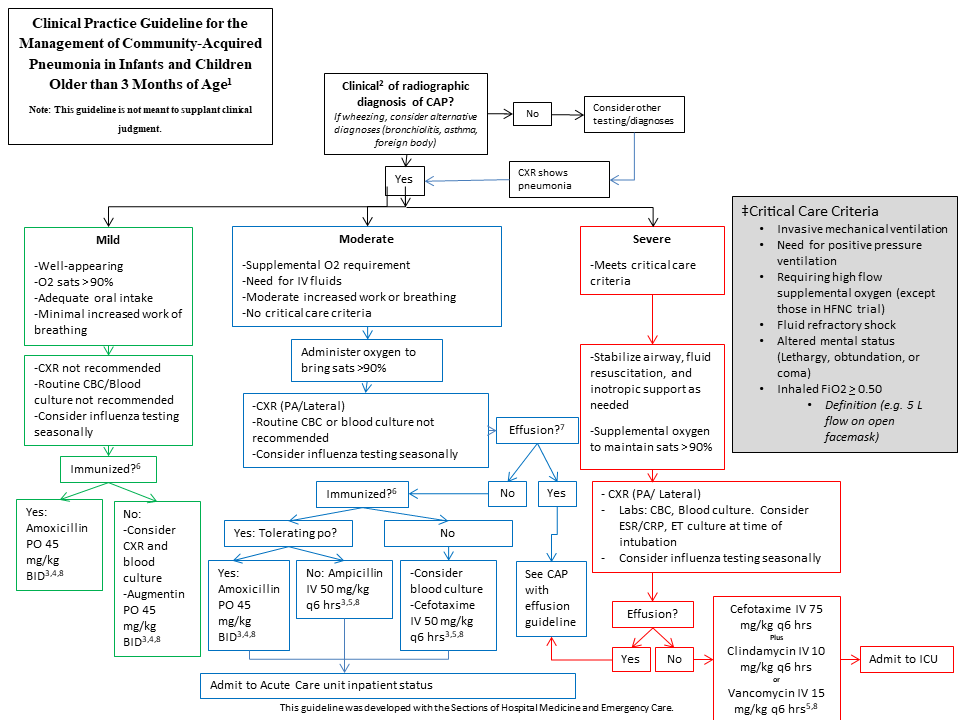

Supplement: SUPPLEMENTARY MATERIAL [file pqs-3-e105-s001.tif]
